# Engagement in Self‐Care Activities among Dementia Caregivers: A Content Analysis of Micro‐Longitudinal Digital Journal Data

**DOI:** 10.1002/alz70858_100490

**Published:** 2025-12-25

**Authors:** Jane Chung, Viviana Le, Emily Mroz

**Affiliations:** ^1^ Emory University, Atlanta, GA, USA

## Abstract

**Background:**

The growing number of people living with dementia (PWD) places increasing demands on family caregivers. Caregivers often neglect their own health, leading to elevated stress and poorer quality of life. Self‐care refers to the activities and practices individuals undertake to maintain quality of life. Self‐care activities are essential for supporting caregivers’ well‐being and self‐efficacy. This qualitative study characterized dimensions of self‐care activities reported by caregivers and explored how they framed these practices within the context of home‐based dementia caregiving.

**Method:**

Ten female family caregivers of PWD participated in the study (age range: 26‐75). We utilized a messenger app‐based journaling technique and micro‐longitudinal design to gather these caregivers’ reports of their self‐care activities every other day over four weeks. Participants generated 137 journal entries. After reading all entries multiple times, we inductively established 14 self‐care dimensions. A content analysis was then conducted to characterize types of self‐care tasks.

**Result:**

Participants reported self‐care activities across 5‐13 dimensions, with activity types and frequency variability. Seven caregivers reported engaging in daily or near‐daily self‐care. The majority of caregivers identified sleep (e.g., “took a nap before work” or “slept in”; *n* = 9) and eating (“ate breakfast” or “stayed on eating plan”; *n* = 9) as typical self‐care activities. Caregivers also reported engaging in physical exercise, attending social gatherings, participating in support groups, taking vitamins, watching TV, taking a shower, and engaging in spiritual activities as forms of self‐care. Interestingly, they also identified managing errands and seeking job opportunities as self‐care activities. Participant responses were classified under three overarching domains: Survival, Self‐Esteem, and Self‐Actualization.

**Conclusion:**

This study sheds new light on the ways caregivers interpret what “counts” as self‐care when reporting on daily self‐care engagement. While participants indicated that they engage in self‐care most days, some activities that they nominated may be rudimentary rather than restorative. Other tasks related to carrying out important personal roles (e.g., as an employee, as an aunt) demonstrate that dementia‐caregivers may interpret opportunities to foster other aspects of their identities as essential self‐care. These findings have implications for new approaches to educate caregivers on self‐care and encourage caregivers to engage in self‐care interventions.

